# Chronic Viral Hepatitis in a Cohort of Inflammatory Bowel Disease Patients from Southern Italy: A Case-Control Study

**DOI:** 10.3390/pathogens9110870

**Published:** 2020-10-23

**Authors:** Giuseppe Losurdo, Andrea Iannone, Antonella Contaldo, Michele Barone, Enzo Ierardi, Alfredo Di Leo, Mariabeatrice Principi

**Affiliations:** 1 Section of Gastroenterology, Department of Emergency and Organ Transplantation, University “Aldo Moro” of Bari, 70124 Bari, Italy; giuseppelos@alice.it (G.L.); ianan@hotmail.it (A.I.); contaldoantonella@gmail.com (A.C.); michele.barone@uniba.it (M.B.); ierardi.enzo@gmail.com (E.I.); b.principi@gmail.com (M.P.); 2 Ph.D. Course in Organs and Tissues Transplantation and Cellular Therapies, Department of Emergency and Organ Transplantation, University “Aldo Moro” of Bari, 70124 Bari, Italy

**Keywords:** inflammatory bowel disease, hepatitis C, hepatitis B, chronic viral hepatitis, liver elastography

## Abstract

We performed an epidemiologic study to assess the prevalence of chronic viral hepatitis in inflammatory bowel disease (IBD) and to detect their possible relationships. Methods: It was a single centre cohort cross-sectional study, during October 2016 and October 2017. Consecutive IBD adult patients and a control group of non-IBD subjects were recruited. All patients underwent laboratory investigations to detect chronic hepatitis B (HBV) and C (HCV) infection. Parameters of liver function, elastography and IBD features were collected. Univariate analysis was performed by Student’s *t* or chi-square test. Multivariate analysis was performed by binomial logistic regression and odds ratios (ORs) were calculated. We enrolled 807 IBD patients and 189 controls. Thirty-five (4.3%) had chronic viral hepatitis: 28 HCV (3.4%, versus 5.3% in controls, *p* = 0.24) and 7 HBV (0.9% versus 0.5% in controls, *p* = 0.64). More men were observed in the IBD–hepatitis group (71.2% versus 58.2%, *p* < 0.001). Patients with IBD and chronic viral hepatitis had a higher mean age and showed a higher frequency of diabetes, hypertension and wider waist circumference. They suffered more frequently from ulcerative colitis. Liver stiffness was greater in subjects with IBD and chronic viral hepatitis (7.0 ± 4.4 versus 5.0 ± 1.2 KPa; *p* < 0.001). At multivariate analysis, only old age directly correlated with viral hepatitis risk (OR = 1.05, 95%CI 1.02–1.08, *p* < 0.001). In conclusion, the prevalence of HBV/HCV in IBD is low in our region. Age may be the only independent factor of viral hepatitis–IBD association. Finally, this study firstly measured liver stiffness in a large scale, showing higher values in subjects with both diseases.

## 1. Introduction

Inflammatory bowel disease (IBD) is a heterogeneous group of inflammatory disorders of the gastrointestinal tract characterized by chronic inflammation of the mucosa, with transmural damage for Crohn’s disease, with the recurrent clinical course [1]. IBD encompasses two major clinical forms, i.e., Crohn’s disease (CD) and ulcerative colitis (UC). Since the treatment of IBD is based in selected cases on immunosuppressive agents (thiopurines and biologic drugs such as monoclonal antibodies), an accurate clinical and laboratory assessment is preliminarily required to look for chronic infections that may have a severe flare under biologic drugs [2]. Among these, chronic viral hepatitis and in particular chronic hepatitis B (CHB) and chronic hepatitis C (CHC) are advised to be investigated by guidelines before starting immunosuppressive treatment [3]. Indeed, all HBsAg positive subjects should start antiviral agents before undergoing biologic treatment to prevent potentially serious hepatitis B flares [3]. According to current guidelines, CHC is not considered as a contraindication to IBD treatment even if it should be considered that immunomodulators could increase viremia thus worsening the progression towards severe liver fibrosis and, therefore, caution is advisable in this context [3]. On the other hand, patients with IBD may suffer from a concomitant liver disorder (primary sclerosing cholangitis, autoimmune hepatitis and overlap syndromes [4,5,6]) or assume drugs (thiopurines) that may provoke liver damage [7]. Additionally, it has been demonstrated that IBD subjects are more prone to develop non alcoholic fatty liver disease [8]. For these reasons, it is important to identify patients with both IBD and chronic viral hepatitis, since the interplay between the management of these conditions may accelerate the progression of liver damage. Previous reports demonstrated a quite variable prevalence of CHB/CHC in IBD, ranging from 6% to 25% [9,10]. Therefore, we aimed to perform an epidemiologic study to assess the prevalence of chronic viral hepatitis in IBD in our geographic area (Southern Italy) and to detect the possible relationship of viral liver infection and specific peculiarities of IBD.

## 2. Results

We totally enrolled 807 subjects with IBD, of whom 438 (54.3%) were affected by CD and 369 (45.7%) by UC. Of these, 35 (4.3%) patients had chronic viral hepatitis, in particular we found 28 CHC (3.4%) and 7 CHB (0.9%). Among the 189 controls, the prevalence of CHB and CHC was 0.5% and 5.3% respectively, not statistically significant compared to the IBD group. Additionally, the control group was similar for age and sex, while we observed a higher number of smokers and subjects with hypertension in the control group. IBD and controls had similar liver stiffness values and fibrosis stage. Even the finding of steatosis did not differ between IBD and controls. Further details are reported in Table 1.

A serology indicative of HBV effective vaccination (anti-HBsAg > 100 mIU/mL) was observed in 232 (28.7%) patients, and vaccinated patients were younger than non-vaccinated ones (43.1 ± 14.1 versus 45.9 ± 16.2 years, *p* = 0.02). Previous contact with hepatitis B virus, landmarked by HBc IgG positivity and HBV DNA negativity, was found in 62 patients (7.7%).

The results of the comparison of demographic and anthropometric data of the two cohorts (IBD with and without viral hepatitis) are summarized in Table 2. Patients with chronic viral hepatitis had a higher mean age and lower frequency of smoking and alcohol consumption. More men were observed in the IBD–hepatitis group (71.2% versus 58.2%, *p* < 0.001). Age at diagnosis was 25.6 ± 6.1 in IBD without hepatitis and 26.9 ± 4.6 in the IBD with the hepatitis group (*p* = 0.21). From a metabolic point of view, these subjects showed a higher frequency of diabetes, hypertension and wider waist circumference. No differences were observed in the lipid and glycemic profile.

The results of the comparison of IBD features of the two cohorts (IBD with and without viral hepatitis) are summarized in Table 3. Subjects with chronic viral hepatitis suffered more frequently from UC. Instead, CD patients with CHC/CHB had undergone less surgical operations; in detail, the 50% (4 out of 8) of patients with surgical procedures performed before 1990 had CHC, while patients undergoing surgery after 1990 did not show any type of viral hepatitis (*p* < 0.001). Furthermore, it was found that patients with CHC/CHB were less frequently exposed to infliximab, antibiotics or probiotics, while no differences were found with other biologic or immunomodulatory drugs. Most of the IBD patients in both groups were in the remission phase, as highlighted by clinical indexes.

In terms of liver function, the two populations were homogeneous, except for the basal liver stiffness, measured by Fibroscan, which was greater in subjects with chronic viral hepatitis than in those without this condition (7.0 ± 4.4 KPa versus 5.0 ± 1.2 KPa; *p* < 0.001). Moreover, patients with advanced fibrosis, i.e., F3 or F4, were significantly more in the hepatitis group (8.6% and 5.7% respectively), than in IBD without hepatitis (1.3% and 0.8%). This subgroup of IBD patients, however, was affected by the following non-viral liver chronic disorders: 5 cases of primary sclerosing cholangitis, 2 autoimmune hepatitis, 2 primary biliary cholangitis and 7 non-alcoholic steatohepatitis. Results are summarized in Table 4.

At multivariate analysis, based on the significant factors at the univariate analysis, only the age directly correlated with the risk of viral hepatitis (OR = 1.05, 95% CI 1.02–1.08, *p* < 0.001).

## 3. Discussion

Chronic viral hepatitis is a worldwide health issue. It is estimated that about 120–200 million people suffer from CHC and 240–350 million from CHB [11], with a mean world prevalence of 5% and 2% for HBV and HCV, respectively [11]. In Italy, a previous study in our region found HCV prevalence of 2.6% in the period 2005–2009 [12]. Furthermore, several studies attempted to assess the epidemiology of chronic viral hepatitis in IBD. Former studies described a prevalence much higher in IBD than in the general population [9,10], while recent evidence [13,14,15,16,17] showed prevalence comparable between the two groups, ranging from 1% to 9%. Herein, we found a prevalence of 3.4% for CHC and 0.9% for CHB, a result that perfectly mirrors the situation in Italy and is in accordance to recent literature reports [13,16,17]. In our series, we demonstrated that the prevalence of HBV and HCV was similar between controls and IBD, and this is a confirmatory finding, since another study from our geographic area has found a similar HCV prevalence of 2.6% [12]. Consequently, we could speculate that IBD may not be associated to increased risk of HCV infection in our region.

It has been hypothesized that previous surgical procedures could be independent risk factors for CHB/CHC [10]. However, our analysis showed a surprisingly opposite result, since subjects with chronic hepatitis experienced less surgical interventions, and this may reflect the evolution of procedures of tool sterilization and knowledge about transmission of viral hepatitis. Indeed, surgical exposure as a risk factor was reported mainly in outdated studies. On the other hand, our analysis demonstrated that only an advanced age was independently associated to increased risk of CHB/CHC (OR = 1.05); it is possible that surgery performed before the diffusion of presurgical hepatitis screening could explain this result, and the fact that CHC were more common in subjects operated before 1990 could be a confirmatory finding. Indeed, the introduction of the HBV vaccine and HCV routine detection allowed an improvement in the prevention measures against viral hepatitis transmission during surgery or blood donation, thus reducing the risk of infection in young generations [18]. As expected, vaccinated patients were younger than non-vaccinated ones, and this may be another possible explanation of our result.

Furthermore, it was found that these patients were less frequently exposed to infliximab, antibiotics or probiotics, while no differences were found with other biologic or immunomodulatory drugs. In this regard, the potential risks of hepatitis reactivation under biologic drugs may have induced a reduction of infliximab prescription, as already suggested by Park et al. [19].

Another significant result was the higher basal liver stiffness in subjects with chronic viral hepatitis than in those without this condition (7.0 ± 4.4 KPa versus 5.0 ± 1.2 KPa; *p* < 0.001). This was confirmed by the higher proportion of F3–F4 fibrosis in the hepatitis group (8.6% and 5.7% respectively), than in IBD without hepatitis (1.3% and 0.8%). We believe that, despite that this was an expected finding, it may represent novel evidence since, at the best of our knowledge, liver elastography has been used only for IBD patients assuming metotrexate [20,21] to monitor drug hepatotoxicity. However, this is the first study that measured systematically liver fibrosis in all IBD subjects.

Another interesting finding at the univariate analysis was that IBD patients with chronic hepatitis suffered more frequently from diabetes, hypertension and were characterized by a large waist circumference. In this perspective, it has been shown that HCV may have a direct atherogenic effect [22] and could promote diabetes by inducing gluconeogenic genes, accumulation of lipids and targeting of lipid storage organelles, as suggested by basic and clinical studies [23,24,25,26,27]. Furthermore, we demonstrated that IBD subjects with CHC/CHB assumed less alcohol and consumed fewer cigarettes, and this event may be explained by the issue that patients with liver disease are frequently advised to stop some voluptuary habits such as ethanol drinking. The prevalence of steatosis is a factor that may overestimate stiffness measurement [28]; however in our cohort its prevalence was similar between IBD with or without hepatitis, therefore a possible bias was ruled out.

The presence of a condition of double chronicity (IBD and concomitant CHC/CHB) may underlie critical issues related to a possible different progression of liver damage. Currently, there are no data available in the literature concerning a more rapid evolution of hepatic fibrosis in this subgroup of patients, although it has been hypothesized that this process may be accelerated by the immunosuppressive therapy employed for IBD [29]. This could be acknowledged as a possible limitation of our study. Since this is a transversal cohort study, we were not able to assess the evolution of liver fibrosis during a follow up period. So, we have no proof to demonstrate such a hypothesis. However, a study is ongoing in our unit aimed to elucidate this topic.

## 4. Materials and Methods

### 4.1. Patients Enrolment

This study was planned as a single center cohort cross-sectional study. In the period October 2016–October 2017 patients followed-up in our IBD outpatient gastroenterology unit (Policlinico di Bari University Hospital, Puglia, Italy) were consecutively recruited, regardless of age. The diagnosis of IBD was performed, according to the current ECCO-ESGAR Guidelines, based on the combination of clinical, laboratory and instrumental data (endoscopy, histology and radiology) [30]. Unclassified IBD were not considered. In the same period of time, a control group of non-IBD patients, matched for age and sex, attending the functional and motor disorders of gastrointestinal system outpatient unit, was enrolled. Enrolled patients provided written informed consent. The study was approved by the independent Ethics Committee of the Policlinico di Bari (protocol no. 4862) and performed according to the statement of the Declaration of Helsinki.

By considering a prevalence of viral hepatitis of 2.6% as reported in our geographic region [12], a precision of 0.05 and a 90% level of confidence, a minimum sample size of 22 patients with chronic viral hepatitis was needed for our purpose. We excluded HIV coinfected patients and subjects who did not give or were unable to provide written informed consent.

### 4.2. Data Collection

For all patients, the following data were collected: clinical history, anthropometric parameters (sex, age, height, weight, body mass index (BMI), smoking, alcohol intake, simultaneous liver disorders other than viral hepatitis and comorbidities) and IBD diagnosis and staging related data (year of first diagnosis, extension/behavior of disease according to the Montreal classification, disease activity using partial Mayo (for clinical UC activity), Harvey Bradshaw index (for clinical CD activity) scores, ongoing and previous therapy and previous surgical procedures) [31,32]. Unfortunately, endoscopic activity scores were not available for all patients, therefore they were not analyzed Then we collected laboratory data performed within 3 months of enrollment: full blood count, creatinine, erythrocyte sedimentation rate (ESR), C-reactive protein (PCR), fecal calprotectin, aspartate transaminase (AST), alanine transaminase (ALT), gamma-glutamyl transpeptidase (GGT), alkaline phosphatase (ALP) and total and conjugated bilirubin. Hepatitis B virus (HBV) markers (in detail HBsAg, anti-HBs and anti-HBc IgG) and hepatitis C (HCV) markers (anti-HCV) were collected. CHB and CHC were diagnosed, respectively, if HBsAg and anti-HCV were positive for at least 6 months. Only for patients who tested positive for HBV and/or HCV chronic infection, determination of HBV-DNA or HCV-RNA was performed. Additionally, we carried out liver ultrasound and liver elastography.

### 4.3. Evaluation of Hepatic Fibrosis Stage Using Fibroscan

Each patient, fasting for at least 12 h, underwent the determination of the liver stiffness by Fibroscan (Echosens, Paris, France) at the time of enrollment. Measurements of the liver stiffness were performed on the right lobe of the liver, on a site localized by previous ultrasound evaluation, through the intercostal spaces with patient in supine position. Ten valid measurements were performed on each patient and only exams with a success rate of at least 60% and an interquartile interval <30% were considered reliable [33]. We excluded from the study conditions that technically prevented the execution of liver elastography (severe obesity, narrowness of intercostal spaces). Liver stiffness values ≥7.0 KPa, 8.7 KPa, 10.3 KPa and 14 kPa were considered as representative of mild (F1), moderate (F2), advanced fibrosis (F3) and cirrhosis, respectively.

### 4.4. Statistic Analysis

The enrolled population was divided into two cohorts: IBD with and without concomitant CHB and/or CHC. The continuous variables were expressed as mean ± standard deviation, while categorical variables as percentages. On univariate analysis, the comparison between continuous variables was performed by a Student’s *t* test, while the chi-square test was used for categorical variables. Multivariate analysis was performed by means of a binomial logistic regression to determine which factors could influence the presence of CHB/CHC in IBD patients. For the independent variables the odds ratios (ORs) were calculated and their 95% confidence interval (CI). All statistical tests were two-tailed and statistical significance was set at OR < 0.05. The statistical analysis was performed using the software SPSS version 23.0 for Windows, Armonk, NY: IBM Corp.

## 5. Conclusions

In conclusion, our study suggests that the age may be the only independent factor of viral hepatitis–IBD association and a possible explanation may be related to HBV vaccination and HCV screening in young people. Moreover, other factors such as biological therapy and metabolic syndrome need to be investigated in prospective studies on large series. As expected, the presence of HBV and HCV are associated to an increased risk of liver fibrosis in patients of IBD.

## Figures and Tables

**Table 1:** Comparison of demographic/clinical characteristics of inflammatory bowel disease (IBD) and non-IBD controls patients.

Variable	IBD (*n* = 807)	Non-IBD (*n* = 189)	*p* Value
**Age, years (mean ± standard deviation)**	46.2 ± 13.2	45.8 ± 17.8	0.73
**BMI, Kg/m^2^ (mean ± standard deviation)**	24.5 ± 5.0	24.4 ± 4.3	0.79
**Male sex, *n* (%)**	474 (58.7)	105 (55.5)	0.42
**Smokers, *n* (%)**	132 (16.3)	45 (23.8)	0.01
**Alcohol intake, *n* (%)** **<10 g/day** **>10 g/day**	90 (11.1) 5 (0.6)	53 (28.0) 0 (0)	0.22
**Diabetes, *n* (%)**	39 (4.8)	7 (3.7)	0.69
**Hypertension, *n* (%)**	79 (17.0)	41 (21.7)	<0.001
Abdominal circumference, *n* (%) *	138 (17.1)	13 (6.8)	<0.001
**Liver stiffness kPa (mean ± standard deviation)**	5.1 ± 2.1	4.9 ± 1.7	0.22
**Fibrosis stage**			0.15
**F0** **F1** **F2** **F3** **F4**	725 (90.0) 52 (6.4) 9 (1.1) 13 (1.6) 8 (0.9)	164 (86.9) 12 (6.3) 6 (3.2) 5 (2.6) 2 (1.0)	
**Ultrasound picture of steatosis, *n* (%)**	231 (28.6)	44 (23.3)	0.14
**Chronic hepatitis B**	7 (0.9)	1 (0.5)	0.64
**Chronic hepatitis C**	28 (3.4)	10 (5.3)	0.24

* Subjects with a value >102 cm for males and >88 cm for females. IBD: inflammatory bowel disease and BMI: body mass index.

**Table 2:** Comparison of demographic, anthropometric and metabolic characteristics of patients with IBD with or without chronic viral hepatitis.

Variable	IBD with Chronic Viral Hepatitis (*n* = 35)	IBD without Chronic Viral Hepatitis (*n* = 772)	*p* Value
**Age, years (mean ± SD)**	57.3 ± 14.5	44.5 ± 15.6	**<0.001**
**Female sex (%)**	28.8	41.8	**<0.001**
**Smokers (%)**	8.6	16.7	**0.001**
**Ethanol assumption (%)** **<10 g/day** **>10 g/day**	11.4 0	11.1 0.6	**<0.001**
**Diabetes (%)**	5.7	4.8	**<0.001**
**Hypertension (%)**	22.8	16.9	**<0.001**
Waist circumference * (%)	28.5	21.6	**<0.001**
**BMI, Kg/m^2^ (mean ± SD)**	23.4 ± 17.1	21.2 ± 11.2	0.36
**Total cholesterol (mg/dL)** **(mean ± SD)**	171 ± 45	177 ± 41	0.59
**HDL (mg/dL)** **(mean ± SD)**	66 ± 38	54 ± 17	0.06
**LDL (mg/dL)** **(mean ± SD)**	101 ± 40	101 ± 34	0.96
**Triglycerides (mg/dL)** **(mean ± SD)**	96 ± 27	112 ± 59	0.30
**Fasting blood glucose (mg/dL)** **(mean ± SD)**	112 ± 59	96 ± 30	0.42

IBD: inflammatory bowel diseases; BMI: body mass index; HDL: high density lipoprotein; LDL: low density lipoprotein. * Subjects with a value >102 cm for males and >88 cm for females.

**Table 3:** Comparison of main IBD features of patients with IBD with or without chronic viral hepatitis.

Variable	IBD with Chronic Viral Hepatitis (*n* = 35)	IBD without Chronic Viral Hepatitis (*n* = 772)	*p* Value
**Crohn’s disease (%)**	45.7	54.6	**<0.001**
**HBI (mean ± SD)**	0.3 ± 0.4	0.2 ± 0.5	0.49
**CD behavior (%)** **B1 inflammatory** **B2 stenosing** **B3 penetrating** **p Perianal**	60 10 30 0	55.6 30.2 14.1 0.7	0.80
**CD localization (%)** **L1 Ileal** **L2 Colon** **L3 Ileocolic** **L4 upper + L3** **L4 + L1**	25 20 25 5 0	24.9 31.3 41.6 1.9 0.3	0.57
**Previous surgery for CD (%)**	8.6	13.7	**<0.001**
**Ulcerative colitis (%)**	54.3	45.4	**0.002**
**Mayo partial score** **(mean ± SD)**	1.7 ± 1.6	1.6 ± 0.9	0.56
**UC localization (%)** **E1 Proctitis** **E2 left colitis** **E3 subtotal/pancolitis**	66.6 26.6 6.6	47.1 16.5 36.4	0.64
**Azathioprine (%)**	25.7	37.6	0.10
**Systemic steroids (%)** **<3 courses/year** **>3 courses/year**	65.7 5.7	58.0 12.8	0.30
**Topical steroids (%)** **In course** **Used previously**	20 14.3	11.4 23.7	**0.03**
**Infliximab (%)**	14.3	18.4	**<0.001**
**Adalimumab (%)**	11.4	16.5	0.20
**Golimumab (%)**	5.7	3.4	0.11
**Biosimilars (%)**	0	1.8	0.06
**Antibiotics (%)**	60	55.7	**0.03**
**Probiotics (%)**	74.3	63.6	**0.008**
**Fecal calprotectin ^#^ (%)**	5.5	5.7	**0.99**
**ESR (mm/h)** **(mean ± SD)**	13.3 ± 13.9	15.1 ± 14.8	0.57
**CRP (mg/dL)** **(mean ± SD)**	4.1 ± 12.6	1.7 ± 5.9	0.09

IBD: inflammatory bowel diseases; UC: ulcerative colitis; CD: Crohn’s disease; HBI: Harvey Bradshaw Index; ESR: erythrocyte sedimentation rate; CRP: C reactive protein; ^#^ >200 µg/g.

**Table 4:** Comparison of characteristics of liver profile in patients with IBD with or without chronic viral hepatitis.

Variable	IBD with Chronic Viral Hepatitis (*n* = 35)	IBD without Chronic Viral Hepatitis (*n* = 772)	*p* Value
**Ethanol assumption (%)** **<10 g/day** **>10 g/day**	11.4 0	11.1 0.6	**<0.001**
**Basal Liver stiffness, kPa** **(mean ± SD)**	7.7 ± 4.4	5.0 ± 1.2	**<0.001**
**Severity of fibrosis, *n* (%)**			**<0.001**
**F0** **F1** **F2** **F3** **F4**	28 (80) 2 (5.7) 0 (0) 3 (8.6) 2 (5.7)	697 (90.3) 50 (6.5) 9 (1.1) 10 (1.3) 6 (0.8)	
**Ultrasound picture of steatosis, *n* (%)**	10 (28.6)	221 (28.6)	0.22
**AST (× ULN)**	0.72 ± 0.41	0.59 ± 0.58	0.17
**ALT (× ULN)**	0.69 ± 0.54	0.77 ± 0.62	0.94
**GGT (× ULN)**	0.45 ± 0.29	0.58 ± 0.93	0.08
**ALP (× ULN)**	0.90 ± 1.21	0.58 ± 0.54	**0.04**
**Total bilirubin (mg/dL)** **(mean ± SD)**	0.79 ± 0.64	0.74 ± 0.64	0.77
**Conjugated bilirubin (mg/dL)** **(mean ± SD)**	0.27 ± 0.12	0.23 ± 0.67	0.47
**Platelets (×10^3^)** **(mean ± SD)**	253 ± 77	265 ± 88	0.46
**APRI score** **(mean ± SD)**	0.41 ± 0.66	0.37 ± 1.79	0.92

IBD: inflammatory bowel diseases; AST: aspartate transaminase; ULN: beyond the upper normality limit; ALT: alanine transaminase; GGT: gamma glutamyl transpeptidase; ALP: alkaline phosphatase; APRI: AST to Platelet Ratio Index.
